# Targeting Mitochondrial Bioenergetics as a Therapeutic Strategy for Chronic Lymphocytic Leukemia

**DOI:** 10.1155/2018/2426712

**Published:** 2018-02-28

**Authors:** Subir Roy Chowdhury, Versha Banerji

**Affiliations:** ^1^Research Institute in Oncology and Hematology, CancerCare Manitoba, Winnipeg, Canada; ^2^Department of Biochemistry and Medical Genetics, Max Rady College of Medicine, Rady Faculty of Health Sciences, University of Manitoba, Winnipeg, MB, Canada; ^3^Department of Internal Medicine, Max Rady College of Medicine, Rady Faculty of Health Sciences, University of Manitoba, Winnipeg, MB, Canada

## Abstract

Altered cellular metabolism is considered a hallmark of cancer and is fast becoming an avenue for therapeutic intervention. Mitochondria have recently been viewed as an important cellular compartment that fuels the metabolic demands of cancer cells. Mitochondria are the major source of ATP and metabolites necessary to fulfill the bioenergetics and biosynthetic demands of cancer cells. Furthermore, mitochondria are central to cell death and the main source for generation of reactive oxygen species (ROS). Overall, the growing evidence now suggests that mitochondrial bioenergetics, biogenesis, ROS production, and adaptation to intrinsic oxidative stress are elevated in chronic lymphocytic leukemia (CLL). Hence, recent studies have shown that mitochondrial metabolism could be targeted for cancer therapy. This review focuses the recent advancements in targeting mitochondrial metabolism for the treatment of CLL.

## 1. Introduction to CLL and Its Treatment

Chronic lymphocytic leukemia (CLL) is part of a spectrum of lymphoproliferative disorders that include monoclonal B-cell lymphocytosis (MBL) and small lymphocytic lymphoma (SLL) which are defined by the aberrant accumulation of mature CD19-/CD5-positive monoclonal B-lymphocytes in peripheral blood [1]. In CLL, monoclonal B-lymphocytes must achieve a threshold of >5 × 10^9^/L in the peripheral blood, whereas in SLL and MBL, they remain lower [1]. The lymph nodes, spleen, and bone marrow may be affected in CLL or SLL. In MBL, there is no evidence of enlarged lymph nodes or spleen and blood counts are normal [2]. The progression rate of MBL to CLL is approximately 1% per year requiring treatment [2]. For the purpose of this review, CLL and SLL will be considered as one entity.

CLL is the most common leukemia in the Western world affecting adult patients, and the true incidence is underestimated [3]. The clinical course of CLL is highly variable, ranging from a long-lasting stable disease requiring observation to a one that rapidly progresses requiring treatment [4]. The natural history of this indolent lymphoproliferative disorder is most patients relapse and require retreatment [5]. Treatments have exploited cell proliferation or DNA replication to target cells within the peripheral blood, lymph nodes, and bone marrow [6]. Common treatments include nucleoside analogues (fludarabine), alkylating agents (cyclophosphamide, chlorambucil, and bendamustine) in combination with monoclonal antibodies directed against CD 20 (rituximab or obinutuzimab) in the front line setting [4]. Within the relapse setting or in high-risk patients harboring a deletion of 17p chromosome, targeted agents such as tyrosine kinase inhibitors, ibrutinib, a BTK (Bruton's tyrosine kinase) inhibitor, and idelalisib, a PI3Kdelta (phosphoinositide-3-kinase) inhibitor, as well as small molecules, specifically venetoclax, a BCL-2 (B-cell lymphoma gene) inhibitor, are currently available [4]. The sites of disease relapse are often within the lymph nodes and bone marrow microenvironments. This is in part due to acquired tumor suppressor loss specifically ATM (ataxia telangiectasia mutated gene) and/or TP53 or the development of clonal evolution [7, 8]. These CLL cells are resilient based on their ability to escape apoptosis and furthermore protected by the tumor microenvironment. This may be as a result of alterations in cellular metabolism which represent a hallmark of cancer [9]. Certain metabolic changes in cells are essential in order for them to be transformed to cancerous cells, and as a consequence, the metabolic framework is substantially altered [10]. Thus, nutrients, cytokines, and signaling molecules within the cancer cell and its microenvironment promote cell and promote drug resistance mainly by crosstalk from the stromal microenvironment or tissue niche that enhances leukemia cell viability [11]. Signaling through direct cell-cell contact, secretion of stromal factors and metabolic interactions of the tissue microenvironment also results in the protection of leukemia cells [12, 13].

Although altered cellular metabolism was recognized as a characteristic of cancer cells by Otto Warburg almost a century ago, it was popularized in the 1950s, only to recently have actual functional links been established between oncogenic pathways and cellular metabolism [14]. Mitochondria play an important role in cellular metabolism. Mitochondria are involved in cell death, cell differentiation, innate immunity, hypoxia, and the metabolism of amino acids, calcium, iron-sulphur clusters including heme biosynthesis [15]. In addition, altered mitochondrial bioenergetics, the redox balance of cells, and proapoptotic factors are controlled by mitochondria that may lead to cell death. Thus, crucial roles of mitochondria in the neoplastic phenotype notably are resistance to apoptosis, uncontrolled proliferation, and metabolic reprogramming [16, 17]. The increasing data eventually indicate that mitochondria may be the prime target for cancer therapy rather than simple bystanders for cancer maintenance.

## 2. Mitochondrial DNA

Mitochondria are important bioenergetics and biosynthetic factories critical for normal cell function and human health [18]. Unlike any other organelle, the mitochondrion has its own DNA which can be altered and result in disease conditions. The human mitochondrial genome is a double-stranded circular structure that is about 16.6 kb pairs in length. It contains 37 genes that code 2 rRNAs, 22 tRNAs, and 13 mitochondrial proteins of the respiratory chain [19, 20]. One of main features that differentiate mitochondrial genome from nuclear genome is the intrinsic susceptibility to damage. Mitochondrial DNA (mtDNA) is substantially more susceptible to mutations than nuclear DNA (nDNA) as it is less protected due to its complex chromatin organization, limited repair capacity, and also ROS generated by the electron transport chain being close to its proximity; however, this remains controversial [21]. Given that there are multiple copies of mtDNA in each cell, mutations can affect either all of them (this is termed as homoplasmy) or a proportion (termed as heteroplasmy). This becomes important when thinking of reasons for why CLL occurs and how this may contribute to the physiology of the disease. However, it is important to point out that mitochondrial proteins involved in oxidative phosphorylation (OXPHOS) and ATP production are vastly encoded by nuclear DNA.

## 3. Mitochondrial Physiology and ROS

**Figure 1:**
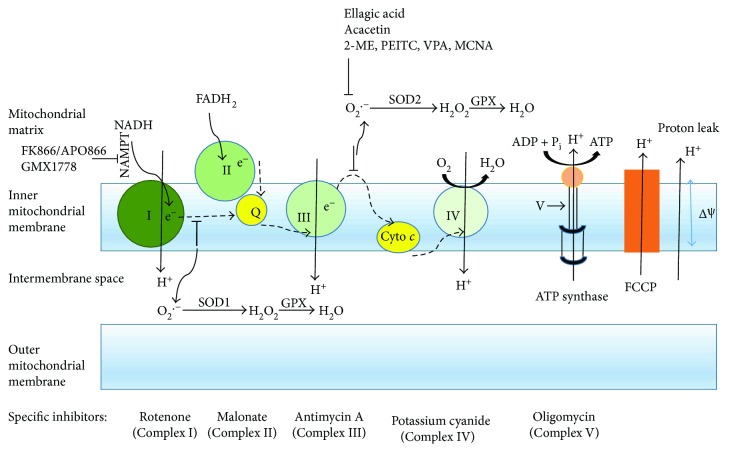
Scheme of the mitochondrial electron transport chain. During the respiration process in mitochondria, electrons from the oxidized state of substrates are transported through a series of electron transport carriers (dashed arrows) located in the inner mitochondrial membrane. Electrons (e^−^) raised from NADH and FADH_2_ enter the electron transport chain at Complexes I and II, respectively. The free energy is released from Complexes I, III, and IV by the gradual decrease of redox potential while electrons are passing and translocating protons (H^+^) from the matrix into the intermembrane space of mitochondria. The proton electrochemical potential gradient generated across the inner mitochondrial membrane is referred as the proton-motive force (pmf). The pmf is used to generate ATP by ATP synthase and also allows the return of protons into the matrix. The redox state of mitochondrial complexes is shown in green. Several chemical compounds (ellagic acid; acacetin; 2-ME, 2-methoxiestradiol; PEITC, *β*-phenylethyl isothiocyanate; VPA, valproic acid; and MCNA, metal-containing nucleoside analogues) alter the ROS generation in CLL. The comparatively darker carrier indicates a more reduced state and vice versa. Cyto *c*: cytochrome *c*; NADH: nicotinamide-adenine dinucleotide (reduced); FADH_2_: flavin-adenine dinucleotide (reduced); Q: ubiquinone; ΔΨ: mitochondrial membrane potential; FCCP: carbonyl cyanide-*p*-trifluoromethoxyphenylhydrazone; I, II, III, and IV attribute to mitochondrial complexes; NAMPT: nicotinamide phosphoribosyltransferase; SOD1 or SOD2: superoxide dismutase 1 or 2; GPX: glutathione peroxidase.

The main physiological function of mitochondria is the production of ATP by OXPHOS and the essential metabolites to accomplish the bioenergetics and biosynthetic demands of normal and cancer cells [22]. Three major and important aspects of OXPHOS involved in mitochondrial pathogenesis are (i) energy production, (ii) ROS production, and (iii) apoptosis [21]. Carbon fuels are utilized by mitochondria to produce ATP. The sources of carbon pools are pyruvate generated from glycolysis, amino acids like glutamine, and fatty acids. The Krebs cycle in the mitochondrial matrix uses these carbon fuels to generate the reducing equivalents NADH and FADH_2_, which subsequently pass their electrons to the electron transport chain (ETC). The transfer of electrons is coupled to the efflux of hydrogen ions from the matrix to the intermembrane space by mitochondrial complexes I, III, and IV (Figure 1). The two main components generated by the proton-motive force are the membrane potential that occurs from the net movement of positive charges across the inner mitochondrial membrane and the pH gradient. Most of the energy largely supplied by the membrane potential (cca 150–180 mV) is reserved in the gradient. Complex V uses this proton-motive force to generate ATP from ADP and P_i_. Thus, the mitochondrial membrane potential is crucial to maintain the physiological function of the ETC in order to produce ATP. A significant loss of mitochondrial membrane potential renders cells depleted of energy with subsequent death. Besides, the generation of NADH and FADH_2_, the Krebs cycle also produces intermediates that can fuel into multiple biosynthetic ways to synthesize glucose, amino acids, lipids, heme, and nucleotides. Hence, mitochondria serve as a center for both catabolic and anabolic metabolism.

ROS is a byproduct of the mitochondrial electron transport chain. Free radicals are mainly generated in the inner mitochondrial membrane during the process of OXPHOS. The leakage of electrons primarily occurs at complexes I and III that leads to partial reduction of oxygen and forms superoxide. Superoxide anions are subsequently and rapidly dismutated to hydrogen peroxide by superoxide dismutases 1 and 2 (SOD1, Cu-Zn superoxide dismutase, and SOD2, Mn-superoxide dismutase). SOD1 is located in the inner mitochondrial membrane space and SOD2 in the mitochondrial matrix. H_2_O_2_ is then converted to water by glutathione peroxidase (Figure 1). The important role of ROS has been implicated in the regulation of growth and survival of cancer as well as structural damages to cells along with lipids, membranes, and DNA [23].

## 4. Mitochondrial Bioenergetics

**Figure 2:**
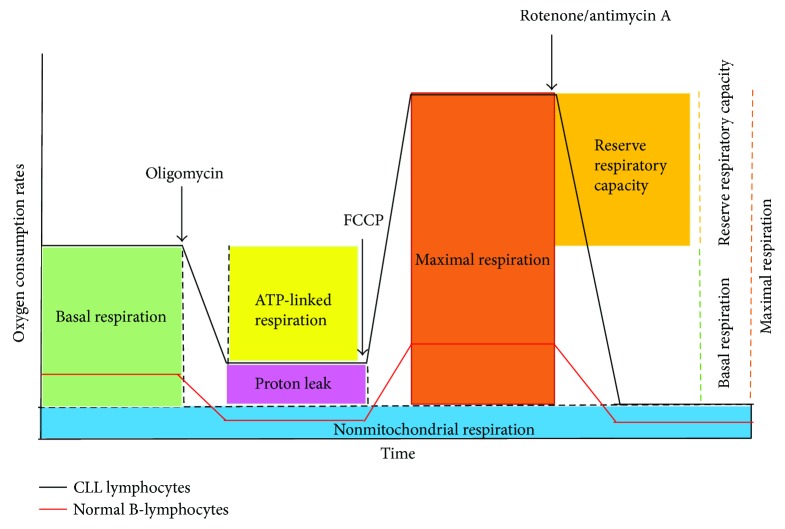
Bioenergetics profile in normal B lymphocytes and primary CLL cells. General scheme of bioenergetics parameters during mitochondrial stress test is shown. Sequential injections of oligomycin, FCCP, rotenone, and antimycin A measure basal respiration (green), ATP-linked oxygen consumption (yellow), proton leak (pink), maximal respiration (orange), reserve respiratory capacity (gold: maximal respiration—basal respiration), and nonmitochondrial respiration (blue). Dashed lines indicate OCR for the portion of each defined parameter. CLL, chronic lymphocytic leukemia lymphocytes, (black line) and normal B-lymphocytes (red line). The comparison of mitochondrial of bioenergetics between CLL and normal B-lymphocytes shown in this figure is adapted based on the results demonstrated by Jitschin et al. [29].

Mitochondrial bioenergetics is important to evaluate the pathogenesis of mitochondrial diseases including cancer. Recently developed new techniques are implemented to quantify mitochondrial function and cellular bioenergetics in order to avoid issues associated with mitochondrial isolation or cell permeabilization [24]. The most commonly utilized bioenergetics parameters (Figure 2), basal respiration, proton leak, coupling efficiency, maximal respiration, respiratory control ratio, reserve respiratory capacity, and nonmitochondrial respirations, are defined herein in intact cells to further understand the bioenergetics profile of mitochondria and its importance [24, 25]. Routine respiration in intact cells is termed as basal respiration, especially by the Seahorse analyzer users. This artificially so-called basal respiration depends on the cellular activity and substrate supplied. Under physiological conditions, cells usually at basal level require only a part of their total bioenergetics capability. The portion of basal respiration inhibited by oligomycin, the ATP synthase inhibitor, can be referred as coupled respiration. The fraction of basal respiration that is not coupled to ATP production is referred to proton leak. Proton leak may forecast mitochondrial injury and may be involved in the regulation of ATP production. The coupling efficiency is calculated by the fraction of oxygen consumption rates driven to produce ATP related to basal respiration. The coupling efficiency varies with ATP demand. The maximal oxygen consumption rate can be achieved by an uncoupler (e.g., FCCP: carbonyl cyanide-*p*-trifluoromethoxyphenylhydrazone). An uncoupler stimulates the respiratory chain to operate at maximum capacity and causes prompt oxidation of substrates from sugar, fat, to amino acid. A titration of an uncoupler is highly recommended in order to achieve the optimum concentration necessary for the maximal stimulation. Single dose of uncoupler in experiments may fail to yield the estimation of maximal respiratory capacity. Respiratory control ratio is defined as the ratio of uncoupled respiration and oligomycin-treated respiration rates. This ratio depends on the substrate oxidation and proton leak; it is not affected by ATP turnover. One of the most important bioenergetics parameters is “reserve respiratory capacity” or “spare respiratory capacity” to evaluate potential respiratory capacity to scope the stressed conditions. It is calculated by the difference between the maximal respiration achieved by an uncoupler and the basal respiration. The respiration rate after the addition of specific inhibitors of mitochondrial complexes I and III, rotenone and antimycin A, respectively, is nonmitochondrial respiration. It is subtracted from other respiration rates in order to evaluate the accurate measure of mitochondrial respiration.

## 5. Mitochondrial DNA and CLL

Through GWAS (genome-wide association studies), susceptibility genes were associated with apoptosis and the mitochondrial outer membrane which are important factors in CLL pathophysiology [26]. Environmental exposure may also play a role in its development [27], yet no true causative agents have been identified. Interestingly, the mtDNA structure of patients with CLL is no different from that of normal individuals; however, it has been shown that an increase in mtDNA copy number is associated with an increased risk for the development of CLL [28]. Treatments may also interfere with mtDNA. The mtDNA analysis of 20 CLL patients revealed that heteroplasmic mutations are significantly more frequent in CLL cells of treated patients compared to untreated [19]. The findings in this study suggest that heteroplasmy mutations caused by the chemotherapy in primary CLL cells are associated with increased ROS generation. This may lead to further development of chemoresistance and frequent relapses given the role of ROS in CLL.

## 6. Mitochondria-Derived ROS and Oxidative Stress in CLL

Tumor cells as well as tumor-associated cells can generate abundant ROS. However, the underlying mechanisms of oxidative stress in cancer patients often remain ambiguous. Mitochondrial respiration rate is increased in CLL cells, and as a result, the levels of mitochondria-derived ROS are higher in CLL cells than in normal B cells, and increased oxidative stress can lead to chemotherapy resistance in CLL cells [29]. A variety of antioxidant defenses, for example, intracellular glutathione, glutathione peroxidase, glutathione transferases, catalase, and superoxide dismutases: cytosolic copper-zinc superoxide dismutase (Cu-Zn SOD) and mitochondrial Manganese superoxide dismutase (MnSOD) control ROS levels which enables cells to regulate normal oxidative stress and to avoid excessive oxidative stress [30]. MnSOD plays an important role in metabolizing superoxides; therefore, the reduced MnSOD expression contributes to increase mitochondrial ROS in CLL cells [29]. Increased ROS through the inhibition of MnSOD was shown to induce CLL cell death [31], while elevated ROS levels promote genetic instability and mobilize cell-signaling [32, 33]. Oxidative stress also attenuates immune responses which in CLL may lead to progressive infectious complications and second malignancies [34]. Numerous recent studies suggested an interrelationship between tumor-specific metabolism and excess of ROS. Tumor cells learn to adapt to permanent oxidative stress. They sustain protective pathways [35] that favor resistance towards anticancer agents [36, 37]. This is key in the behavior of CLL cells and their ability to escape the benefits of current chemotherapeutics. It also enables novel strategies to be employed in the treatment of this incurable disease [10]. Recent studies suggest that CLL cells adjust to their increased energy demands by increasing their mitochondrial activity. Jitschin et al. found that the number of mitochondria (demonstrated by electron microscopy), the total mitochondrial mass, mitochondrial biogenesis, mitochondrial bioenergetics (basal, maximal, and ATP-linked respiration rates), mitochondrial membrane potential, and mitochondria-derived ROS and oxidative stress are increased in CLL cells compared to normal B-lymphocytes [29]. There is possibility that certain drugs can further enhance mitochondrial ROS and thereby overwhelm the cancer cells' protective systems that could selectively impact CLL cells opposed to normal B-lymphocytes.

## 7. Importance of Mitochondria in CLL Cell Survival and Microenvironment

The survival of a cell depends on its ability to meet its energy requirements. Metabolic imbalances and augmented resistance to mitochondrial apoptosis are characteristics of cancer cells. Even though there has been recent progress in the understanding of molecular mechanisms in CLL, this disease still remains incurable. Therefore, it is necessary to pinpoint more elements that exclusively favor cell survival in order to target them. The importance of mitochondrial biogenesis and OXPHOS system has not been fully assessed yet. Subunits of mitochondrial complexes encoded by mtDNA are crucial to maintain function of OXPHOS. Otto Warburg, the famous German scientist, introduced the hypothesis that cancer cells depend overwhelmingly on glycolysis rather than OXPHOS for survival [38]. The glycolytic inhibitors as a therapeutic target to control cell proliferation in various cancers were not successful, even though malignant cells are highly glycolytic. These findings suggest that the Warburg hypothesis is not applicable to all malignancies. However, recent findings revealed that some tumors remarkably rely on OXPHOS for survival including CLL [29, 39, 40]. Therefore, OXPHOS may be the potential therapeutic target in order to arrest the uncontrolled proliferation of malignant cells.

The microenvironment for CLL cells is defined as the interactions between stromal cells and matrix [41]. The communication between CLL and the microenvironment affects survival and proliferation of CLL cells and drug resistance that may be conferred by the remaining disease after treatment. Even though drugs for CLL therapy available in the market are efficient in killing cells *in vitro*, the therapeutic efficacy rapidly declines *in vivo* due to the presence of stromal cells [11]. Since the survival of CLL cells is affected by mitochondrial metabolism, mitochondria may have a role on the conditions of the microenvironment in CLL cells and contribute to drug resistance. The study performed by Li et al. identified perhexiline, a carnitine palmitoyltransferase inhibitor that abolishes the transport of fatty acid into mitochondria and selectively kills CLL cells in the presence of bone marrow stromal cells and *in vivo* [42]. This demonstrates that by altering mitochondrial metabolism, one can impact CLL cell survival and its interactions within the microenvironment.

## 8. List of Therapeutic Compounds Targeting Mitochondrial Bioenergetics, Redox Pathways, and Cell Survival in CLL

Since CLL cells have an increased mitochondrial biogenesis, such as increased mitochondrial mass and number, membrane potential, ATP production, mitochondrial DNA copy numbers, mitochondrial bioenergetics profile, and ROS, these changes provide a possibility to preferentially target CLL cell mitochondria to improve therapeutic selectivity [19, 28]. Recent findings are described below (Table 1).

The polyphenolic compound, *ellagic acid* selectively leads to apoptosis mediated by ROS overproduction in CLL cells that directly targets mitochondria [43]. The antioxidant and antiproliferative properties have been found in several *in vitro* and small animal models [44, 45]. Ellagic acid can induce apoptosis while increasing ROS production, mitochondria swelling, decrease in MMP resulting in cytochrome *c* release, caspase 3 cleavage, and apoptosis in CLL cells.

*Sodium dichloroacetate (DCA)* exhibits anti-CLL activity and is synergistic with the p53 activator nutlin-3 [46]. DCA showed a dose-dependent anti-CLL effect in both primary CLL and CLL-like cell lines with a functional p53. At the molecular level, DCA, via posttranscriptional modifications of p53 protein and in the presence of nutlin-3, increased expression of p53-target genes, particularly p21. Genetic silencing of p21 significantly rescued the DCA + nutlin3-induced cell death phenotype. This study substantiates that DCA needs to be further evaluated as a potential therapeutic agent for CLL, likely in combination with other compounds. CLL cells often acquire defects in p53 status and this becomes a common mechanism of drug resistance, and the ability to enhance and maintain p53 function would enable standard chemotherapeutics, a continued role in CLL.

*Acacetin (4*′*-methoxy-5,7-dihydroxyflavone)*, a natural flavone, can selectively induce apoptosis in CLL cells by directly targeting mitochondria through increased ROS production, loss of MMP, mitochondrial permeability transition pore, release of cytochrome *c*, and caspase 3 activation, while non-CLL lymphocytes remain unaffected [47]. Oral administration of acacetin showed potent anticancer activity in CLL xenograft mouse models. This compound is attractive because it does not belong to other classes of drugs that are currently utilized in CLL therapy, and as a result, it may be beneficial as mechanisms of resistance emerge with novel agents in CLL.

The antidiabetic drug *metformin* was found to inhibit the mitochondrial respiratory chain and consequent OXPHOS in human epithelial type 2 (HeP2) cells originated from human laryngeal carcinoma and 143B cells from human bone osteosarcoma [48, 49]. Metformin decreases tumor growth indirectly, that is, systematic effect; it lowers glucose and insulin or directly inhibits energetic metabolism and cellular pathways involved in proliferation through AMPK-dependent [50, 51] or AMPK-independent mechanisms [52, 53]. This has also been studied in CLL, where metformin-induced apoptosis in resting CLL cells and inhibition of cell cycle entry when CLL cells were stimulated by CD40-CD40L ligation (a mimic of the CLL microenvironment), while non-CLL lymphocytes remained unaffected at the same doses [54]. This arrest in cell cycle was accompanied by decreased expression of proteins associated with survival and proliferation and inhibition of signal transduction pathways responsible for CLL progression as well as loss of intracellular glucose available for glycolysis. Given the common use of metformin in patients in general, metformin alone would not be effective as a treatment for CLL in doses that would be tolerable by patients. However, in drug combination experiments with fludarabine or the BCL2 inhibitor ABT-737, metformin enabled lower doses of each agent studied to decrease the apoptotic threshold and potentiate CLL cell death [54].

*2-methoxiestradiol (2-ME)*, a reagent that inhibits superoxide dismutase, induces apoptosis in leukemia cells by free radical-mediated mechanism [55]. The cellular production of O_2_^−^ is necessary for the antileukemia activity of 2-ME *in vitro*. Primary patient CLL cells demonstrate heterogeneous levels of cellular O_2_^−^; however, leukemia cells from previously treated patients had higher O_2_ levels. As newer mechanisms of resistance and challenging tocxicities evolve with some of the newer agents, alternative mechanisms of cell death need to be explored. Interestingly, CLL cells with high levels of superoxide were more sensitive to 2-ME, and thus this may be an attractive option for those patients who have failed previous therapies. In addition, CLL cells could be sensitized by the use of exogenous ROS-generating agents, such as arsenic trioxide in CLL cells with a low level of endogenous superoxide which were resistant to 2-ME to significantly enhance antileukemia activity. Thus, combination of ROS-producing agents and SOD inhibitors may provide a new strategy to enhance therapeutic activity and overcome drug resistance in CLL patients who have or have not been previously treated.

Fludarabine is the standard treatment for younger patients with CLL. At the same time, fludarabine resistance is a common clinical dilemma. Fludarabine-resistant CLL cells can be eliminated by *β-phenylethyl isothiocyanate (PEITC)* through a redox-mediated mechanism [56]. However, sensitivity to PEITC was observed in fludarabine-resistant and fludarabine-sensitive cells while non-CLL lymphocytes were not. Exposure of CLL cells to PEITC causes severe glutathione depletion, increased ROS, and oxidation of mitochondrial cardiolipin leading to cell death. This study demonstrates that PEITC via a redox-mediated mechanism eliminates fludarabine resistance with minimal toxicity to normal lymphocytes. Given that current fludarabine-based treatments remain the standard of care in young, fit, low-risk patients, this agent warrants further clinical evaluation to reduce toxicity and improve efficacy of fludarabine-based regimes.

*FK866/APO866*, a nicotinamide phosphoribosyltransferase (NAMPT) inhibitor, mediates apoptosis in CLL cells [57]. NAMPT is overexpressed in CLL cells versus non-CLL lymphocytes and thus an attractive target for CLL-specific cell death. FK866 induces CLL cell death by depleting cellular NAD^+^ content at 24 hours along with loss of MMP, ROS increase, and induction of apoptotic signaling within 48 hours. These on-target effects were confirmed by NAD-mediated rescue of NAD and ATP loss, apoptotic signaling, and viability. Patients who had previously been treated with fludarabine were sensitive to FK866, and fludarabine and FK866 were synergistic at clinically relevant concentrations. This paper suggests that FK866 enhance efficacy and/or allow dose reduction of standard chemotherapeutics for improved tolerability. APO866 increases leukemia cell death of cyclosporine-A by inducing mitochondrial and endoplasmic reticulum stress [58]. The combination of APO866 with Pgp (P-glycoprotein-1) inhibitors resulted in a synergistic combination in leukemia cells, while sparing normal blood cells. Combining Pgp inhibitors with APO866 lead to increased intracellular APO866 levels, compounded NAD^+^ and ATP storage, and induced ΔΨm dissipation. This suggests that selectively targeting NAMPT, an enzyme upregulated in malignant B cells, offers an avenue to utilize and repurpose current treatment strategies and potentially reduce toxicities for patients. The challenge with NAMPT inhibitors in patients has been drug delivery by intravenous infusion opposed to oral ingestion. A second oral NAMPT inhibitor, GMX1778 is depicted along with FK866/APO866 at the site of complex I in Figure 1. This may improve the uptake of this agent moving forward.

*Valproic acid (VPA)*, a HDAC (histone deacetylase) inhibitor, increases cell death in CLL-like cell lines. VPA improves fludarabine-induced apoptosis mediated by ROS and involved decreasing AKT and ATM activation in B-cell lymphoid neoplastic cells [59]. This increased apoptosis resulted in the release of cytochrome *c*, activation of caspases, and increased ROS generation. Combination of VPA with fludarabine treatment decreased both phosphorylated and total levels of AKT and ATM both key proteins in CLL signaling and DNA damage, respectively. VPA reduces ATM levels and induced ROS-dependent cell death via the mitochondrial apoptotic pathway when combined with fludarabine. This suggests that HDAC inhibitors could potentiate fludarabine-based treatments while limiting toxicity.

*Venetoclax (ABT-199)* is a BH3 mimetic and a specific inhibitor to BCL2 that is currently approved for treatment of CLL by the U.S. Food and Drug Administration (FDA) for use in CLL patients who have received prior therapy [60, 61]. Since BCL-2 is overexpressed in CLL, it blunts activation of the mitochondrial pathway to apoptosis and is thus required for CLL survival [62]. Venetoclax binds to BCL2, thereby dislodging proapoptotic proteins, BAX and BAK from their binding to BCL-2. This recently approved drug further demonstrates the importance of mitochondrial metabolism. This agent also has high rate of complete responses, and 5% of study patients achieved minimal residual disease negativity [63]. This occurs rarely with tyrosine kinase inhibitors, further pointing to the importance of the mitochondria in CLL.

*ZGDHu-1 [N,N*′*-di-(m-methylphenyi)-3,6-dimethyl-1,4-dihydro-1,2,4,5-tetrazine-1,4-dicarboamide]* is a proteasome inhibitor and has been reported to exhibit antitumor activity [64, 65]. A study has recently been conducted to assess whether this drug has a synergistic effect with fludarabine and mediates apoptosis in CLL cells [66]. CLL cell-specific apoptosis occurred through the mitochondrial pathway as normal cells remained unaffected. Most importantly, a combination of ZGDHu-1 and a sublethal dose of fludarabine led to synergy. Notably, the rate of apoptosis caused by ZGDHu-1 alone or in combination with fludarabine did not correlate with high-risk disease features. Therefore, the use of ZGDHu-1 alone or in combination with fludarabine may further enhance treatment options for CLL patients. This becomes important when thinking of fludarabine-based therapies as it may improve the efficacy and lower the toxicity profile of this drug.

Increased oxidative stress and altered mitochondrial metabolism in CLL cells are associated with the lymphoid oncogene TCL-1 (T cell leukemia 1). Prinz et al. demonstrated that organometallic nucleosides *(MCNA, metal-containing nucleoside analogues)* induce nonclassical cell death that is mitochondrial ROS dependent and facilitated by TCL1 oncogene overexpression [67]. MCNA induced cell death through PARP that was nonautophagic and nonnecrotic as well as caspase- and P53-independent. The authors investigated how these aberrant redox characteristics and bioenergetics of CLL are impacted by TCL1 and how targeting it could be exploited for therapy in the future. The TCL-1 transgenic mice have been characterized by Johnson et al. as a suitable animal model for preclinical drug assessment tool in human CLL [68]. These animals expressed relevant therapeutic targeted proteins with wild-type p53 status and showed sensitivity *in vitro* to therapeutic agents generally used in the treatment of CLL. This also enables a suitable model for mitochondrial targeting in CLL.

ROS inducible *DNA crosslinking agents* are activated aromatic nitrogen mustards, the ability to crosslink DNA. DNA inter-strand crosslinks are identified as one of principle mechanisms for the cytotoxic effect of many antitumor drugs. These compounds exhibit very powerful crosslinking abilities in the presence of H_2_O_2_ and provide a novel strategy for tumor-specific damage. Primary CLL samples were more sensitive (40–80% apoptosis) than non-CLL lymphocytes from healthy donors [69]. They further demonstrated that these compounds function through ROS-dependent mechanisms as NAC rescued the viability effect and decreased ROS generated in primary CLL cells. The data described in this study provide an additional selective agent for development in CLL.

## 9. Conclusion

Mitochondrial metabolism has only now become of interest in the realm of cancer therapy. CLL is a disease that has many mitochondrial metabolic dependencies. However, the nature of metabolic networks that enable abhorrent cell proliferation, cell-cell communication, evasion of apoptosis, and drug resistance remains poorly understood. The list of therapeutic compounds described in this review implements a strong suggestion that targeting mitochondrial bioenergetics and metabolic alterations may provide a mechanistic explanation for the growth advantage and apoptotic resistance of tumor cells. In CLL patients, standard chemi-immunotherapy and novel targeted agents continue to lead to treatment failures. In order to best target this cancer, a multipronged treatment strategy such as combinations with mitochondrial targeting agents and currently approved treatments may enable a chance at cure for a currently incurable cancer.

## Figures and Tables

**Table 1:** List of compounds targeting mitochondrial metabolism in CLL.

Compound	Target	Possible mechanism	References
Ellagic acid (EA, 2,3,7,8-tetrahydroxy-chromeno[5,4,3-cde]chromene-5,10-dione)	Antioxidant and antiproliferative properties (inhibition of DNA binding of certain carcinogens)	↓ MMP, ↑ cytochrome *c* release, caspase 3 activation, and apoptosis	[43]
Sodium dichloroacetate	Pyruvate dehydrogenase kinase	P53 activity	[46]
Acacetin (4′-methoxy-5,7-dihydroxyflavone)	Unknown	↓ MMP, ↑ cytochrome *c* release, caspase 3 activation, and apoptosis	[47]
Metformin (1,1-dimethylbiguanide hydrochloride)	Energetic metabolism, cell proliferation through AMPK-dependent and independent mechanism	Apoptosis, inhibition of cell cycle entry	[54]
2-Methoxiestradiol (2-ME)	Superoxide dismutase inhibition	Apoptosis	[55]
*β*-phenylethyl isothiocyanate (PEITC)	Glutathione antioxidant system	↓ Glutathione, ↑ ROS, oxidation of cardiolipin	[56]
FK866/APO866	NAMPT inhibition	NAD depletion, ↓ cell viability, ↑ ROS	[57, 58]
Valproic acid (VPA)	Histone deacetylase inhibition	↓ AKT and ATM activation ↑ ROS, ↑ Cytochrome *c* release, activation of caspases	[59]
Venetoclax (ABT-199)	BH3 mimetic, BCL-2-selective inhibitor	Apoptosis	[61]
ZGDHu-1 [N,N′-di-(m-methylphenyi)-3,6-dimethyl-1,4-dihydro-1,2,4,5-tetrazine-1,4-dicarboamide]	Proteasome inhibitor	Apoptosis	[66]
MCNA, metal-containing nucleoside analogues	PARP-mediated cell death	↓ OCR, rapid membrane depolarization	[67]
ROS inducible DNA crosslinking agents	DNA crosslinking coupled with H_2_O_2_	Cytotoxic, tumor-specific damage	[69]

↓: decreased; ↑: increased; MMP: mitochondrial membrane potential; ROS: reactive oxygen species; NAMPT: nicotinamide phosphoribosyl transferase; AMPK: 5′ adenosine monophosphate-activated protein kinase; AKT: RAC-alpha serine/threonine-protein kinase; ATM: ataxia telangiectasia mutated; BCL2: B-cell lymphoma gene 2; BH3: BCL2 homology domain 3; PARP: poly (ADP-ribose) polymerase; OCR: oxygen consumption rate.
